# Seroprevalence of Influenza A(H5N1) Virus in Domestic Cats at Epicenter of Dairy Cattle Outbreaks, California, USA, 2024–2026

**DOI:** 10.3201/eid3209.260785

**Published:** 2026-09

**Authors:** Ian G. Bemis, Ismaila Shittu, Jacob F. Gomez, Esther A. Fadaka, Sofia Perez, Gregory C. Gray, Kristen K. Coleman

**Affiliations:** University of Maryland School of Public Health, College Park, Maryland, USA (I.G. Bemis, E.A. Fadaka, S. Perez, K.K. Coleman); University of Maryland College of Agriculture and Natural Resources, College Park (I.G. Bemis, K.K. Coleman); University of Texas Medical Branch Division of Infectious Diseases, Galveston, Texas, USA (I. Shittu, G.C. Gray); Cross Street Small Animal Veterinary Hospital, Tulare, California, USA (J.F. Gomez)

**Keywords:** avian influenza virus, influenza, viruses, H5N1, cross-species transmission, zoonoses, cats, One Health, California, United States, dairy cattle

## Abstract

We conducted a serologic study of domestic cats near the epicenter of the dairy cattle outbreaks of influenza A(H5N1) in California, USA. Three of the 12 cats sampled within 2 km of farms had neutralizing antibodies against H5N1 virus. Proximity to dairy farms was a statistically significant risk factor for seropositivity.

Highly pathogenic avian influenza A(H5N1) clade 2.3.4.4b is emerging in domestic cats (*Felis catus*), presenting urgent public health and animal welfare implications. Historically, cats have not been widely considered important hosts of influenza viruses. However, sporadic infections and outbreaks of avian influenza viruses in cats have occurred for >20 years (*1*). The emergence of H5N1 clade 2.3.4.4b virus has resulted in widespread mammalian infections (*2*,*3*), including an alarming increase in infections among domestic cats, in which the estimated case-fatality rate is 90% (*1*). Although most of those infections emerged from direct bird-to-cat transmission, several recent outbreaks among US cats have involved infected dairy farms (*4*–*6*) or links to workers in the dairy industry (*7*). Contaminated raw dairy milk (*5*,*8*) and raw meat–based diets (*9*) have been identified as sources of infection in cats.

Like swine, cats have α-2,6- and α-2,3-linked sialic acid receptors (*10*) and can be co-infected with avian and human influenza viruses. Thus, with widespread H5N1 virus infections, a growing potential exists for influenza virus co-infections in domestic cats that could result in viral reassortment and selection for novel human-adapted viruses. Infections resulting from cat-to-human transmission of avian influenza have been documented 3 times in the United States, including in a veterinarian and an animal shelter worker exposed to shelter cats infected with influenza A(H7N2) virus in New York, New York, in 2016 (*11*,*12*) and in a veterinary professional exposed to a domestic cat infected with H5N1 virus in Los Angeles, California, in 2024 (*13*). Suspected human-to-cat transmission of H5N1 virus also occurred in 2024 in Michigan, resulting in the deaths of 2 house cats (*7*). Ten additional cats died from another H5N1 outbreak in 2024 in South Dakota (*14*) in which cattle-to-bird-to-cat transmission was suspected.

Although several US dairy industry–related outbreaks of H5N1 among cats have been reported, the resulting seroprevalence of H5N1 virus among domestic cats near affected dairy farms is unknown. Therefore, we performed a serosurvey of domestic cats at the epicenter of the 2024–2025 dairy cattle outbreaks in California’s Central Valley.

## The Study

An animal welfare organization in Tulare County, California, provided serum samples collected from cats during routine blood draws. Because serum samples from routine blood draws were provided, an ethics review was not required. We shipped aliquots to the University of Maryland (College Park, MD, USA) and the University of Texas Medical Branch (Galveston, TX, USA) and tested them using ELISA and H5 microneutralization assays (Appendix). We compared approximate age, sex, weight, and distance to nearest dairy farm between seropositive and seronegative cats (Appendix).

**Figure 1:**
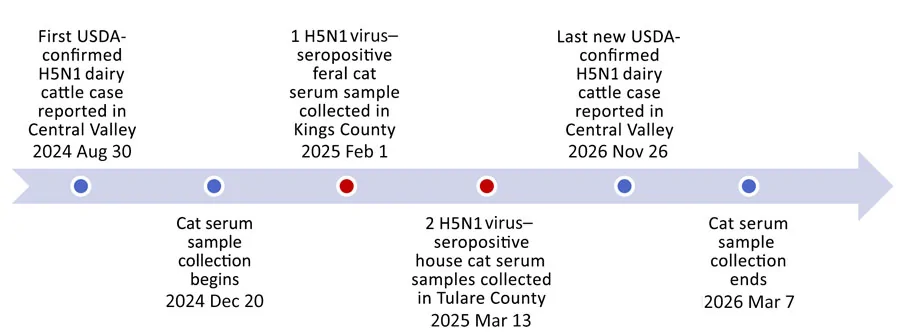
Timeline of highly pathogenic avian influenza A(H5N1) dairy cattle outbreaks and domestic cat (*Felis catus*) serum sample collection, Central Valley, California, USA, 2024–2026. USDA, US Department of Agriculture.

During December 2024–March 2026, we collected 73 serum samples from indoor and outdoor cats (Figure 1) within 9.7 km (6 miles) of >1 dairy farms (mean 4.7 km [2.9 miles]). Among the 73 cats, 3 tested positive for both influenza A virus nucleoprotein antibodies and neutralizing antibodies against H5 clade 2.3.4.4b. One indoor-only and 1 outdoor feral cat had neutralizing antibody titers of 1:160, and 1 indoor-only cat had a titer of 1:640, indicating strong positivity and 100% agreement between the ELISA and microneutralization assay. All negative samples had titers of <1:20.

**Figure 2:**
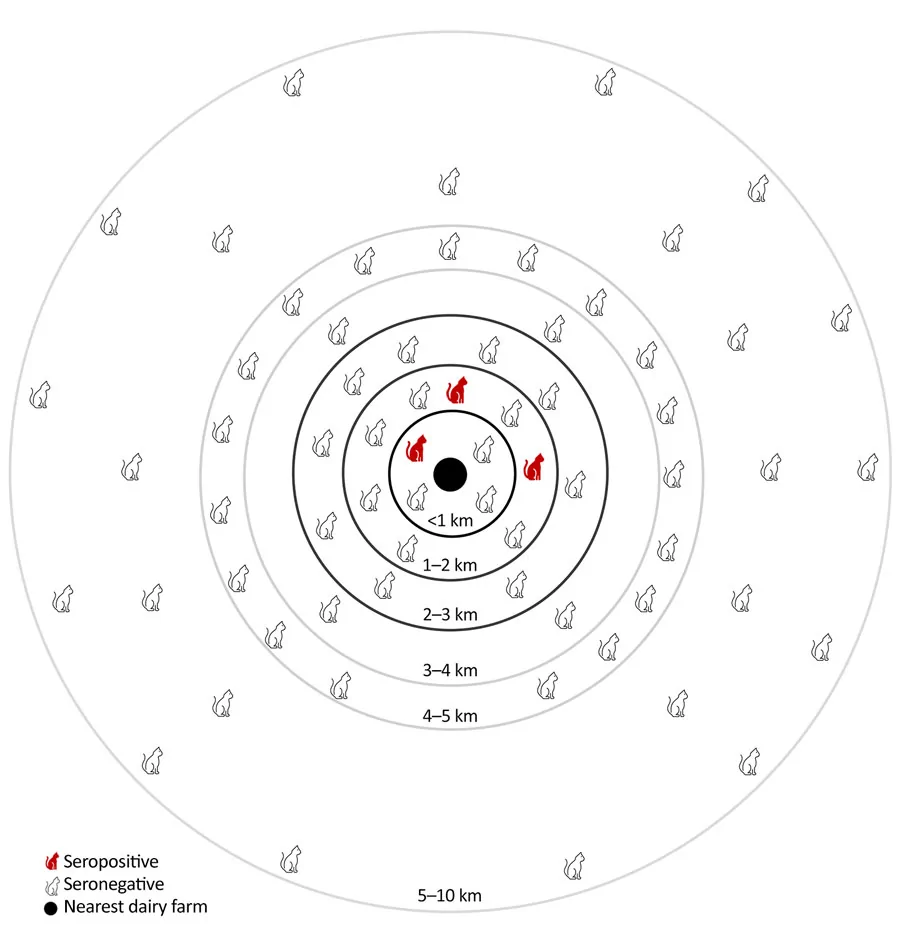
Grouped distance to the nearest dairy farm for domestic cats (*Felis catus*) seropositive and seronegative for highly pathogenic avian influenza A(H5N1) clade 2.3.4.4b virus near epicenter of dairy cattle outbreaks, Central Valley, California, USA, 2024–2026.

**Figure 3:**
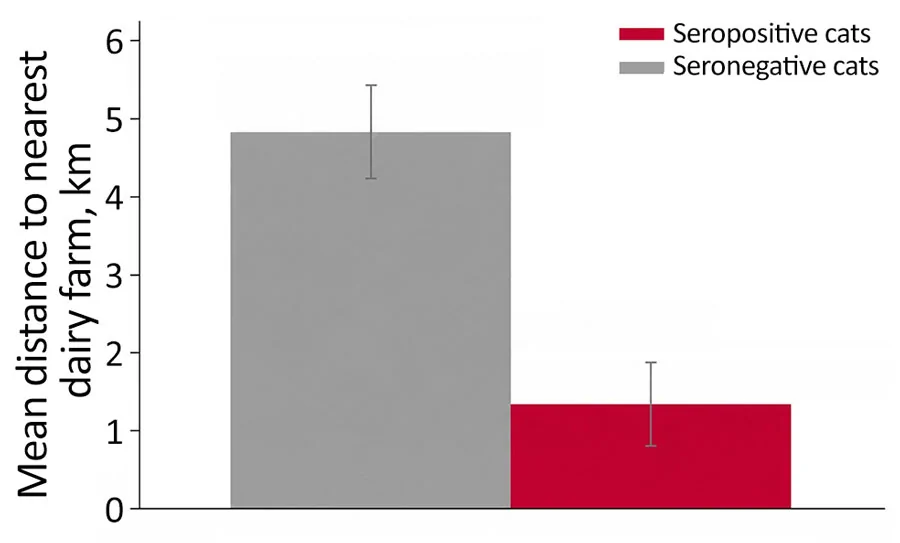
Mean distance to the nearest dairy farm for domestic cats (*Felis catus*) seropositive and seronegative for influenza A(H5N1) clade 2.3.4.4b virus near epicenter of the dairy cattle outbreaks, Central Valley, California, USA, 2024–2026. Error bars represent SEM.

Three (25%) of the 12 cats sampled within 2 km (1.24 miles) of dairy farms were seropositive for H5N1 virus (Figure 2), yielding an overall seropositivity rate of 4.1% for all surveyed cats. Seropositive cats had a mean distance to the nearest dairy farm of 1.34 km (0.8 miles), compared with 4.83 km (3 miles) for seronegative cats (p = 0.014) (Figure 3). Average age of surveyed cats was ≈1.6 years (range ≈3 months–4.9 years); 60% were female and 40% male. Age and sex were not significantly different for seropositive and seronegative cats. Of the 73 cats surveyed, 68 (93%) were in rural areas and 5 (7%) in urban areas. All seropositive cats were in rural areas. No indoor cats were fed raw meat or dairy.

## Conclusions

Avian influenza A(H5N1) clade 2.3.4.4b is an emerging threat to domestic cats and public health. We report a 25% seropositivity rate for the virus among domestic cats within 2 km (1.2 miles) of dairy farms in the epicenter of the H5N1 cattle outbreaks in California during 2024–2026. Proximity to dairy farms in counties experiencing outbreaks may be a risk factor for H5N1 spillover to indoor and outdoor domestic cats. Our research provides insight into potential H5N1 virus sources and transmission routes among domestic cats in the United States, for which data are scarce. Our results may help refine public health guidance on the prevention of avian influenza in cats and set the stage for future studies.

Although only 3 seropositive cats were identified in our study, their closer proximity to dairy farms compared with seronegative cats is an important epidemiologic finding. Although we cannot determine the source (or sources) of H5N1 among the seropositive cats in our study, their close proximity to dairy farms suggests that cats may share a source of infection with infected cattle or that dairy herds are directly infecting nearby cat populations. Infections and deaths also have been reported among cats exposed to H5N1 on affected dairy farms in Texas (*4*), New Mexico (*5*), and Minnesota (*6*) and among domestic cats near dairy farms in South Dakota (*14*) and in the households of Michigan dairy industry workers (*7*). Similar to the South Dakota outbreak (*14*), cattle-to-bird-to-cat transmission is one plausible explanation for the seropositive feral cat identified in our study. The feral cat was found within 0.8 km (0.5 miles) of a farm and could have wandered and been exposed directly to cattle or contaminated milk or other sources. Cat movement onto influenza-infected farms should be studied, in addition to cat exposure to infected rodents and other small mammals. Regarding the seropositive indoor-only cats in our study, both were from a household of a dairy industry worker, similar to the infections in Michigan cats (*7*). The route of transmission, including human-to-cat transmission, cannot be confirmed in our study. However, we can rule out raw meat or dairy as a potential source.

Because of the high case-fatality rate in cats with H5N1 virus infection (*1*), the seroprevalence reported in our study probably is an underestimate of the actual prevalence of H5N1 virus infection in cats in this region. In addition, cats infected with H5N1 virus often have onset of acute encephalitis with multifocal necrosis, resulting in paralysis, blindness, ataxia, and other severe long-term sequelae, sometimes requiring ambulatory assistive devices (*8*). Such complications may preclude working cats from escaping predation or effectively hunting or deterring rodents on farms. To help mitigate that animal welfare and farm maintenance issue, vaccinating dairy cattle should be considered (*15*) because it could protect cattle and other at-risk animals on or near farms.

Our study highlights a critical gap in companion animal surveillance and might be helpful to health agencies as they seek approaches to mitigating this emerging infectious disease threat. Proximity to dairy farms was a risk factor for H5N1 spillover to cats in our study. However, cattle-to-cat transmission of H5N1 virus is a relatively new phenomenon, and most cat infections reported in the literature over the past 20 years have been from bird-to-cat transmission (*1*). To assess the in situ risk for human-adapted reassortant influenza viruses emerging in cats, future work is needed to characterize the seroprevalence of human influenza viruses in cats and avian influenza viruses in cats near outbreaks in poultry and wild birds. Cat surveillance studies also could provide insight into nearby H5N1 outbreaks that have yet to be discovered, fully investigated, or contained.

Appendix Additional information about seroprevalence of influenza A(H5N1) virus in domestic cats at epicenter of dairy cattle outbreaks, California, USA, 2024–2026.
