# Supplementary material for: Seroprevalence of Influenza A(H5N1) Virus in Domestic Cats at Epicenter of Dairy Cattle Outbreaks, California, USA, 2024–2026
Source: Emerg Infect Dis. 2026 Sep;32(9):1474–7. doi: 10.3201/eid3209.260785 (PMC13555714; doi:10.3201/eid3209.260785)
Supplement: Supplementary file 1 [file 26-0785-Techapp-s1.pdf]

Article DOI: https://doi.org/10.3201/eid3209.260785

EID cannot ensure accessibility for supplementary materials supplied by authors. Readers who have difficulty accessing supplementary content should contact the authors for assistance.

Seroprevalence of Influenza A(H5N1) Virus in Domestic Cats at Epicenter of Dairy Cattle Outbreaks, California, USA, 2024–2026

Appendix

Supplemental Materials and Methods

Laboratory Methods

We tested samples upon arrival and stored unused sample material in a −80°C freezer. We tested sera at the University of Maryland School of Public Health using a commercial blocking ELISA (IDEXX AI MultiS-Screen Ab Test; Westbrook, ME) to identify samples containing antibodies reactive to influenza A virus nucleoprotein (NP). We used a sample-to-negative (S/N) ratio of ≤0.5 to determine seropositivity. We tested sera for neutralizing antibodies against influenza A(H5N1) clade 2.3.4.4b at the University of Texas Medical Branch One Health Laboratory using a recombinant H5 microneutralization assay using a BSL-2 compatible mouse adapted PR8 virus pseudotyped to H5 clade 2.3.4.4b as previously described (*1*). We used a minimum dilution of 1:40 as a cutoff for H5 seropositivity. Results of both assays were blinded.

Data Collection and Statistical Analysis

Our study captured two distinct types of cats: feral and indoor-only. Feral cats were part of the trap-neuter-release (TNR) program run by the animal welfare organization that provided the serum samples. Indoor cats were strictly indoor-only and were confirmed to not have outdoor access. For younger feral cats, cat age was estimated using body size, weight, and dental eruption patterns. For older feral cats with skeletal maturity, age estimates relied primarily on dental wear

and calculus accumulation. Because of these limitations, our reported age estimates should be considered approximations rather than precise determinations.

We used a two-tailed t-test to compare the mean cat age and distance to the nearest dairy farm for seropositive and seronegative cats, and a two-tailed Fisher's Exact Test to compare the proportion of seropositive and seronegative cats among males and females. Data analysis was performed using Stata/IC 15.1 (StataCorp, College Station, TX).

Reference

1. Shittu I, Silva D, Oguzie JU, Marushchak LV, Olinger GG, Lednicky JA, et al. A One Health investigation into H5N1 avian influenza virus epizootics on two dairy farms. Clin Infect Dis. 2025;80:331–8. PubMed https://doi.org/10.1093/cid/ciae576
